# Predictive value of persistent NS1 antigen positivity beyond 3rd day for dengue haemorrhagic fever in Sri Lankan children

**DOI:** 10.1186/s13104-019-4250-z

**Published:** 2019-04-08

**Authors:** Mihira Manamperi, Bernard Deepal Wanniarachchi Jayamanne, Thilini Somaratne, Natasha Perera, LakKumar Fernando

**Affiliations:** 1 District General Hospital, Gampaha, Sri Lanka; 2 Department of Public Health, Faculty of Medicine, University of Kelaniya, PO.Box. 06, Thalagolla Road, Ragama, Sri Lanka; 3 Medical Research Institute, Colombo 08, Sri Lanka; 4 Centre for Clinical Management of Dengue and Dengue Haemorrhagic Fever (CCMDDHF), District General Hospital Negombo, Negombo, Sri Lanka

**Keywords:** Dengue NS1, Persistent NS1 positivity, DHF prediction, NS1 predictive ability

## Abstract

**Objective:**

Dengue haemorrhagic fever (DHF) is a major public health concern responsible for significant morbidity in both adult and paediatric populations in Sri Lanka. This study examined if persistent non structural protein 1 (NS1) antigen positivity beyond day 3 was predictive of the occurrence of dengue haemorrhagic fever. The patients were followed up during their in-hospital stay and the severity of the illness was classified according to the WHO classification. The NS1 antigen test was repeated after day 3 of the onset of illness, at least 2 days after the initial test.

**Results:**

One hundred and fifty-seven patients were enrolled. Persistent NS1 antigen test positivity after day 3 of the illness was not predictive of subsequent development of DHF. Out of multiple other demographic and illness related factors assessed, only having a secondary dengue infection was associated with a high risk of DHF (relative risk = 3.077, 95% CI 1.361, 6.954). Persistent NS1 positivity on day 3 may not be indicative of disease severity. However results need to be confirmed by a larger study with quantitative NS1 testing.

**Electronic supplementary material:**

The online version of this article (10.1186/s13104-019-4250-z) contains supplementary material, which is available to authorized users.

## Introduction

Dengue infection has become a major public health concern in Sri Lanka. According to the statistics published by the Epidemiology Unit, 185,688 dengue cases were reported in Sri Lanka during 2017 with 440 deaths [1].

The overall dengue incidence in Sri Lanka was 865.9 per 100,000 population and the case fatality ratio was 0.24 in 2017 [1]. Dengue infection has two different clinical entities: dengue fever and dengue haemorrhagic fever (DHF). Dengue haemorrhagic fever is responsible for the morbidity and mortality observed with the infection. The detection of non-structural protein 1 (NS1) antigen in the serum in the early stage of the disease has become an effective tool to diagnose dengue infection. This study examined if a positive NS1 test on day 3 of fever (or later) could be a predictor of dengue haemorrhagic fever than dengue fever.

NS1 antigen testing is a popular method to diagnose dengue fever in the first 1–3 days since the onset of fever. Since the presence of the antigen indicates viraemia, we hypothesized that patients continuing to show viraemia later in the infection are more likely to have an adverse outcome than in patients who are Non Structural protein 1 antigen negative at a comparable stage of the illness. There are a number of studies in literature that had assessed the effectiveness of NS1 antigen for timely diagnosis of dengue fever, but none had correlated persistent NS1 antigen positivity with disease severity. This investigation is relatively expensive and it is available both in the private sector and certain government hospitals. It can also give a qualitative as well as a quantitative result. Though the latter version is preferable, only the qualitative version of the test was available to conduct the study with the objective of assessing the predictive ability of NS1 antigen positivity on day 3 or beyond of the illness for dengue haemorrhagic fever.

## Main text

### Ethical considerations and approval

Ethical approval was obtained from the ethical review committee, Sri Lanka College of Paediatricians. Strict anonymity was maintained for all data obtained, and any identifying information was kept entirely confidential and removed once the analysis was completed.

### Study design and setting

This prospective study was carried out on NS1 positive patients on day 1 of fever to day 2 of fever, who admitted to the General Paediatric ward and Centre for Clinical Management of Dengue and Dengue Haemorrhagic Fever (CCMDDHF) in District General Hospital Negombo, Sri Lanka from 1st of January 2017 to 31st December 2017. The NS1 antigen test was repeated after day 3 of the onset of illness, at least 2 days after the initial test. We recruited patients up to the age of 18 years.

### Sample size calculation

Sample size was calculated to according to the formula on proportions [2], [Where, n—required sample size, value at 95% significant level = 2, p—expected prediction (assuming 50% of day 01 NS1 positive patients will be positive for day 3 NS1 test), d—confidence limit = 8%], a minimum of 151 sample size was required.

After adding 5% correction the required number of patients will be 151 + 8 = 159. Sampling method was convenience sampling.

### Inclusion criteria for the study

NS1 positive dengue patients between days 1 and 2 since the onset of fever, following informed written parental or legal guardian’s consent were enrolled. They were recruited from the General Paediatric ward and Centre for Clinical Management of Dengue and Dengue Haemorrhagic Fever (CCMDDHF) in the Negombo District General Hospital, Sri Lanka during the period of study. Day 3 considered from 60 h of onset of fever to 84 h of onset of fever (approximately two and half days to three and half days.)

### Exclusion criteria for the study

NS1 negative patients that were diagnosed to have dengue based on serology or clinically, diagnosed dengue patients who never had a NS1 antigen test and those who refused to consent to the study.

### Final diagnosis and DHF grading

Detailed diagnosis on grading of Dengue was carried out by trained physicians and was reviewed in detail by the unit in-charge consultant physician before final confirmation.

### Data collection

All cases corresponding to the above inclusion and exclusion criteria within the study period were enrolled and data were was extracted using an interviewer administered data extraction sheet.

### Data analysis

The SPSS software (Statistical Package for Social Sciences) version 22 was used for statistical analysis. Summary statistics are reported with measures of central tendency and dispersion. Significant correlations were assessed with the Chi square test. Level of statistical significance was set at p < 0.05.

### Results

**Fig. 1:**
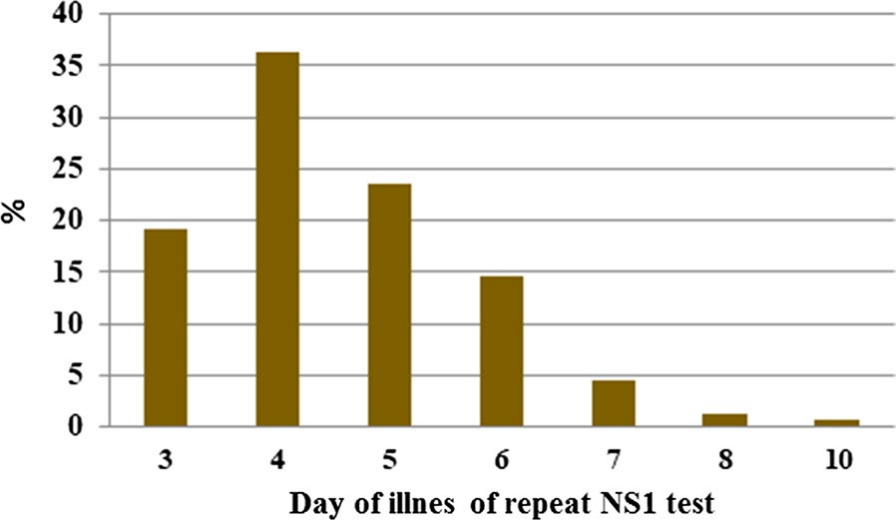
Day of illness of 2nd NS1 test and percentage

One hundred and fifty-seven patients were recruited (81 males, 51.6%, mean age: 10.44 years, range 1–18 years). Of the recruited patients, 61.8% (97/157) had their first NS1 test done on day 1 of fever while 33.8% had their NS1 done on day 2 of fever. Repeat NS1 testing was performed from day 3 onwards up until day 10 of fever. In this regard 19.1% (30/157) had their second NS1 test done on day 3 while 36.3% (57/157) patients had it done by day 4. The repeat NS1 testing percentages for days 5, 6, 7 represented, respectively, 23.6%, 14.6% and 4.5% (Fig. 1).

Repeat NS1 testing was positive in 82.8% (130/157) of patients. Out of the study population 51.6% (81/157) were managed as dengue fever and 48.4% (76/157) were diagnosed as dengue haemorrhagic fever. Six patients out of the 157 subjects (3.8%) had a past history of dengue.

There was no statistically significant association between subjects, who remained NS1 positive beyond day 3 and developed dengue haemorrhagic fever; and those who remained NS1 positive beyond day 3 and developed dengue fever (p value = 0.4697) (Table 1).

**Table 1:** NS 1 status and DF/DHF status

NS1 status at day 3 or beyond	Managed as (DF/DHF)	
	DF	DHF
Positive	66	64
Negative	15	12
Total	81	76

When considering the DHF grading among the patients whose repeat NS1 antigen was positive, 49 of them had a DHF grade I and 13 had a DHF grade II. Two patients were diagnosed to have dengue haemorrhagic fever grade IV and both remained NS 1 positive beyond day 3 of illness which persisted beyond day 8 of illness. All the DHF patients who were detected to be NS1 negative after day 3 were found to be in DHF grade I classification. Since the majority of patients were of DHF grade I and grade II (which is clinically more or less similar), it was not possible to correlate DHF grade with persistent NS1 positivity (Additional file 1: Figure S1).

Thirty-three patients of the study population were tested for both IgG and IgM antibodies. Dengue IgG positivity was a significant predictor of DHF (p = 0.001) (Table 2) while IgM positivity was not a predictor of DHF.

**Table 2:** IgG status vs DF/DHF status

IgG status	Managed as (DF/DHF)	
	DF	DHF
Positive	5	15
Negative	10	3
Total	15	18

The data was also analysed to detect a relationship between gender, blood group and the Rhesus group for patients with and without DHF. We also compared the mean differences in age, body weight and duration of fever between dengue fever and dengue haemorrhagic fever groups. There were no statistically significant differences in any of the above comparisons (Additional file 2: Table S1).

This study has shown an incidental finding where two patients developed haemophagocytic lymphohistiocytosis (HLH) and liver failure and remained NS1 antigen positive even at day 8 of fever.

### Discussion

Dengue infection has become a major public health concern which is responsible for significant morbidity and mortality in both adult and paediatric populations in Sri Lanka. Early diagnosis of the disease and meticulous monitoring has been the key to reduce the number of deaths. According to the latest available statistics published by the epidemiology unit Sri Lanka, there have been 7258 dengue cases which were reported island wide up until February 2019 [1]. Therefore studies that help early diagnosis of dengue fever help to reduce the morbidity and mortality. NS1 antigen testing is a more sensitive method of early detection of dengue fever. Numerous studies have shown the effectiveness of this tool to detect early infection and a persistently high level of NS1 antigen in the blood reflects severe disease [3, 4]. However our study was designed to see if persistent NS1 antigenaemia was associated with having dengue haemorrhagic fever than dengue fever.

Contrary to the established belief, the study did not show a correlation between persistent NS1 antigen positivity (days 3–7 of illness) and DHF or the severe disease with complications. Interestingly, other demographic and illness related factors assessed also did not show an association with DHF except the presence of a secondary infection (When a patient exposed to the dengue infection for the second time or reinfection) can lead to subsequent dengue haemorrhagic fever rather than primary infection (first dengue infection). The latter observation however, is not a novel observation and is partially explained by the hypothesis of “original antigen sin” as proposed by Halstead [5].

Given that the sample size was met, the results though negative are reliable when DHF is taken as a single entity (without subgroup classifications, such as grade I, II, III and IV). It has been hypothesized by others that plasma leakage in dengue haemorrhagic fever, which is the stepping stone to other profound complications such as shock, is driven more by host factors rather than viral factors. The partial immunity obtained by previous infection from a different serotype induces formation of non-neutralizing heterotrophic antibodies that induce the innate and adaptive immune systems to damage the vascular endothelium via cytokine production. The intensity of this response may not be dependent on the level of viraemia once the process is initiated and hence may explain the lack of correlation between NS1 antigenaemia (a viral protein) and the outcome of infection.

Besides, this study was done in a centre of excellence which uses unique fluid management coupled with meticulous monitoring. Therefore, though we had 79 DHF patients none of them went into DHF grade III or IV; if they did, it would reflect suboptimal fluid management during the critical phase. The two patients who were already diagnosed as DHF grade IV, were transferred from another peripheral hospital. Thus, the WHO classification of severity grading of DHF (grades I to IV) was not helpful to correlate with positivity of second NS1 test. Furthermore, the unit applied certain management principles, such as maintaining a pulse pressure of 30 Hgmm by using a relatively low fluid rate which is most of the time static from the onset of leaking. This is hypothesized to control and reduce the rate of leaking. As a result with controlled minimal leaking, patients would not go into shock or impending shock such as grade III and grade IV of DHF. The severity of leaking could be more precisely assessed by the height of the pleural effusion, degree of ascites, bleeding manifestations, pulse pressure and elevation of ALT/AST rather than be confined to the WHO grading system.

### Conclusions and recommendations

Persistent NS1 positivity on day 3 and beyond may not be a predictor of DHF or severe disease. However confirmation of these preliminary results would require a prospective study with quantitative NS1 testing and a larger sample size to accommodate an adequate number of patients to achieve more specific results.

## The limitations

Ideally the NS1 testing should have been performed at regular and fixed time intervals. Unfortunately, as the study was not independently funded, testing could not be done systematically as planned by the investigators and happened on an “as needed” basis during patients’ management. Furthermore the NS1 testing done in this study was a qualitative one. A quantitative assessment would have enabled correlation of antigen titre and better resolution of results (possibility of different outcomes for patients with very high or very low titres). Currently a second study is planned with independent funding and quantitative NS1 testing that will circumvent many of the limitations observed in this study.

## Additional files

**Additional file 1: Figure S1.** NS1 status on day 3 and beyond vs DHF grade.

**Additional file 2: Table S1.** Comparison of means of age, weight and duration of febrile phase with DF/DHF status.

## Abbreviations

CCMDDHF: Centre for Clinical Management of Dengue and Dengue Haemorrhagic Fever
DF: dengue fever
DHF: dengue haemorrhagic fever
HLH: haemophagocytic lymphohistiocytosis
NS1: non structural protein 1
SPSS: Statistical Package for Social Sciences
