# Supplementary figures and images for: Predictive value of persistent NS1 antigen positivity beyond 3rd day for dengue haemorrhagic fever in Sri Lankan children
Source: BMC Res Notes. 2019 Apr 8;12:214. doi: 10.1186/s13104-019-4250-z (PMC6454706; doi:10.1186/s13104-019-4250-z)

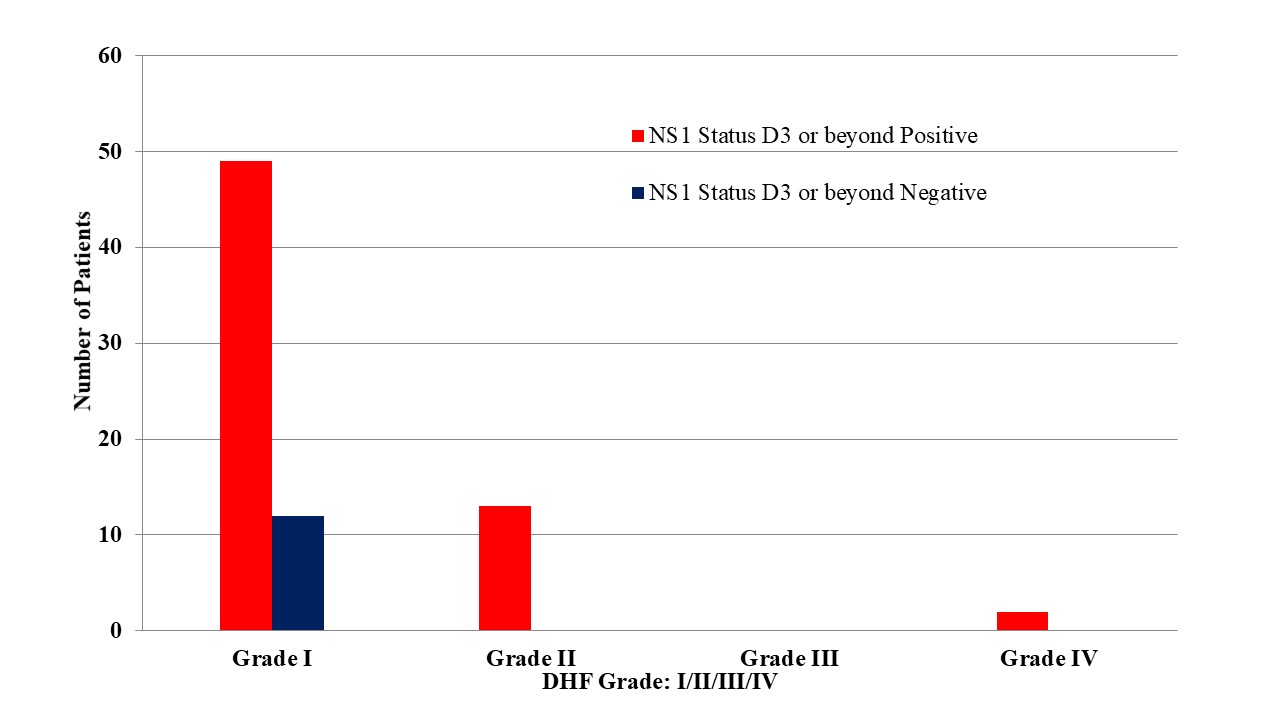

Supplement: Supplementary file 1 — Additional file 1: Figure S1. NS1 status on day 3 and beyond vs DHF grade. [file 13104_2019_4250_MOESM1_ESM.jpg]
