# Supplementary material for: Predictive value of persistent NS1 antigen positivity beyond 3rd day for dengue haemorrhagic fever in Sri Lankan children
Source: BMC Res Notes. 2019 Apr 8;12:214. doi: 10.1186/s13104-019-4250-z (PMC6454706; doi:10.1186/s13104-019-4250-z)
Supplement: Supplementary file 2 — Additional file 2: Table S1. Comparison of means of age, weight and duration of febrile phase with DF/DHF status. [file 13104_2019_4250_MOESM2_ESM.docx]

Additional file 2: Table S1: Comparison of means of age, weight and duration of febrile phase with DF/DHF status

| **Factor** | **Statistic** | **DF** | **DHF** | **P value** |
| --- | --- | --- | --- | --- |
| Age on admission: (years) | Mean | 10.01 | 10.91 | >0.05 |
|  | SD | 4.18 | 4.49 |  |
| Weight (kg) | Mean | 28.38 | 31.93 | >0.05 |
|  | SD | 12.17 | 13.25 |  |
| Febrile phase days : | Mean | 5.84 | 6.20 | >0.05 |
|  | SD | 1.34 | 1.86 |  |
